# Optimal Protocols and Management of Clinical and Genomic Data Collection to Assist in the Early Diagnosis and Treatment of Multiple Congenital Anomalies

**DOI:** 10.3390/children10101673

**Published:** 2023-10-10

**Authors:** Heui Seung Jo, Misun Yang, So Yoon Ahn, Se In Sung, Won Soon Park, Ja-Hyun Jang, Yun Sil Chang

**Affiliations:** 1 Department of Pediatrics, Kangwon National University Hospital, Kangwon National University School of Medicine, Kangwon 24289, Republic of Korea; 2 Department of Pediatrics, Samsung Medical Center, Sungkyunkwan University School of Medicine, Seoul 06351, Republic of Korea; 3 Cell and Gene Therapy Institute, Samsung Medical Center, Seoul 06351, Republic of Korea; 4 Department of Pediatrics, CHA Gangnam Medical Center, CHA University, Seoul 06135, Republic of Korea; 5 Department of Laboratory Medicine and Genetics, Samsung Medical Center, Sungkyunkwan University School of Medicine, Seoul 06351, Republic of Korea; 6 Department of Health Sciences and Technology, Samsung Advanced Institute for Health Sciences and Technology, Sungkyunkwan University, Seoul 06351, Republic of Korea

**Keywords:** congenital anomalies, newborns, whole genome sequencing analysis, protocol, phenotype, registry

## Abstract

Standardized protocols have been designed and developed specifically for clinical information collection and obtaining trio genomic information from infants affected with congenital anomalies (CA) and their parents, as well as securing human biological resources. The protocols include clinical and genomic information collection on multiple CA that were difficult to diagnose using pre-existing screening methods. We obtained human-derived resources and genomic information from 138 cases, including 45 families of infants with CA and their parent trios. For the clinical information collection protocol, criteria for target patient selection and a consent system for collecting and utilizing research resources are crucial. Whole genome sequencing data were generated for all participants, and standardized protocols were developed for resource collection and manufacturing. We recorded the phenotype information according to the Human Phenotype Ontology term, and epidemiological information was collected through an environmental factor questionnaire. Updating and recording of clinical symptoms and genetic information that have been newly added or changed over time are significant. The protocols enabled long-term tracking by including the growth and development status that reflect the important characteristics of newborns. Using these clinical and genetic information collection protocols for CA, an essential platform for early genetic diagnosis and diagnostic research can be established, and new genetic diagnostic guidelines can be presented in the near future.

## 1. Introduction

Congenital anomalies (CA) are one of the main causes of newborn and infant deaths [1,2]. The congenital abnormalities-specific mortality rate was estimated at 2.169/1000 live births among neonates in 2015 [1]. Overall, there were 0.3–2.8/1000 live births of infant deaths due to CA (WHO health Organization) and deaths due to CA (19 European countries) in 2005–2009 [3]. Regional and national differences in mortality rates, as well as underestimations, should be considered [1,3].

The adjusted US national birth prevalence estimates for 29 major birth defects varied from 0.62 to 19.93 per 10,000 live births according to each major defect, based on the 2010–2014 birth cohort [4]. Between 2008 and 2014, the overall prevalence of major birth defects in South Korea was 446.3 per 10,000 births [5]. Genetic causes, including single-gene defects and chromosomal abnormalities, have been identified in at least 25% of CA [6]. With the development of cytogenetic and molecular techniques, the identification of genetically related sites that were previously undiagnosed has increased. Efforts to identify environmental and genetic risk factors for causal determination have become crucial [7]. Sufficient research resources, including in-depth data on patients and parents, as well as human-derived materials, are essential to elucidate risk factors and genetic factors associated with CA.

Rare diseases are characterized by a population of less than 40–50 cases/100,000 individuals or conditions with unknown prevalence due to difficulties in diagnosis [8]. Over 80% of rare diseases are genetic or congenital disorders that manifest during early childhood [9,10]. Most of these diseases are serious or disabling, with a significant economic burden, as they often lack effective treatments or are associated with high-cost therapies. The National Institutes of Health (NIH) Undiagnosed Diseases Program, now expanded nationwide as the Undiagnosed Diseases Network, is led by the NIH and aims to assist in patient diagnosis and treatment decisions. Patients provide medical information such as photographs, imaging data, and biopsy samples, and collaborate with NIH clinical centers to facilitate further diagnostic and treatment decisions. Approximately 47% of patients in the program were pediatric patients, primarily with CA and neurological disorders [11]. South Korea implemented the Rare Disease Management Act, Implementation Decree, and Enforcement Rules in 2016. Through the Rare Disease Registration and Statistics Project and Genetic Diagnosis Support Project, programs for the diagnosis of undiagnosed rare diseases have been developed and implemented [12].

With recent advancements in next-generation sequencing (NGS), personalized medical care tailored to individual patients has become possible for conditions that were previously difficult to diagnose and analyze, enabling more effective treatments [13,14]. This study aimed to establish a diagnostic research foundation for multiple CA through clinical, epidemiological, and genomic information collection protocols.

## 2. Methods/Design

### 2.1. Patient Registry Establishment and Selection of Eligible Patients

**Figure 1:**
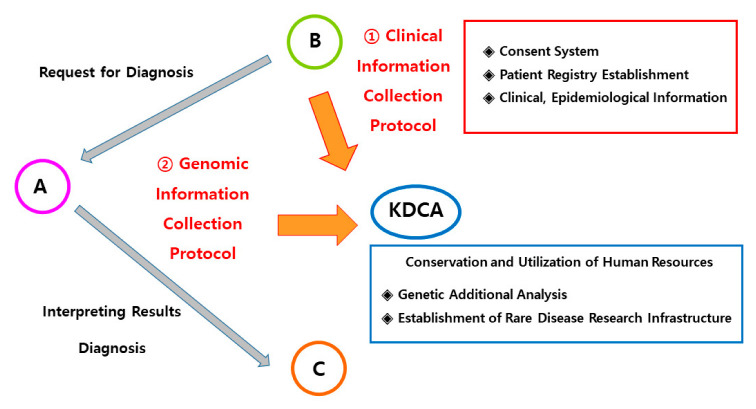
Overview of the study: collection of clinical and genomic information. A: Analysis/Assay of genetic information; B: Babies with undiagnosed genetic diseases; C: Counseling/Control of genetic problem; KDCA: Korea Disease Control and Prevention Agency.

**Figure 2:**
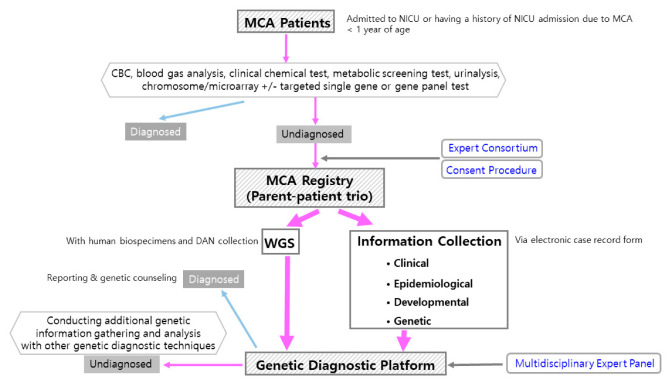
Flow diagram of the study protocol for the registration, data collection, and genetic diagnostic workup for infants with multiple congenital anomalies. MCA Patients: patients who have multiple (two or more) congenital anomalies; CBC: complete blood count; WGS: whole genome sequencing.

This study was developed through two academic research and development projects conducted by the Korea Disease Control and Prevention Agency from 1 April 2021 to 31 December 2022 (Project No. 2021-ER0706-00, 1 April 2021–31 March 2022, CM Project 2021; Project No. 2022-ER0503-00, 28 March 2022–31 December 2022, CM Project 2022) and the established protocol was applied to another ongoing project (CM Project 2023; Project No. 2023-ER0703-00). The study was conducted on living neonatal patients at Samsung Medical Center (SMC) during the study period. An overview of the study is shown in Figure 1 and the protocol is shown in Figure 2.

We targeted newborns with major multiple CA who were negative for all items based on existing conventional test results. The tests included complete blood count, clinical chemical test, blood gas analysis, urinalysis, newborn screening for congenital metabolic disorders, chromosomal analysis, and microarray analysis. Those who had negative results for all items or positive findings that could not explain the newborn phenotypes of multiple CA were invited to participate in this study. In recent rapidly advancing medical environments, there has been an increasing trend of performing targeted single gene testing or gene panel testing based on the phenotype expressed by the newborn when there is clinical suspicion of involvement of specific genetic regions. Therefore, participation in this study was limited to cases where the results of single gene testing or gene panel testing were negative or inconclusive in explaining the newborn’s phenotypes of multiple CA from a medical perspective.

More than two neonatologists in charge of CA newborns discussed the potential participation of the newborns in this genetic study, and the research manager or officer made the final decision regarding suitability. The overall contents of the study were presented to the parents, and a consent form was prepared for those who voluntarily wished to participate. A total of 45 families (135 individuals), including newborns with CA and their parent trios, were selected for participation in the study. The inclusion of other relatives for genetic testing within the family was determined selectively through expert meetings when medically necessary.

### 2.2. Establishment of Consent System

A consent system for the collection and utilization (third-party provision) of human biological materials and related information (clinical, epidemiological, and genomic) was established only when both parents consented to participate in the study. The study protocols were approved by the Institutional Review Boards (IRBs) of the SMC (IRB codes: 2021-04-189, 2022-04-054, 2023-04-057). Prior to the commencement of the study, the research team held meetings and extensive discussions to determine the items and scope of clinical/epidemiological and genomic information to be collected. The appropriateness of each item was submitted to the IRB for ethics review, following a strict ethical review process. The recommendations from the IRB were duly incorporated into the consent form. Furthermore, the researchers underwent education on the collection, production, and donation of human resources conducted by the National Central Human Resources Bank/Biobank Division.

The key elements of the consent form included voluntary participation, purpose/methods/procedures of the study, anticipated risks and discomfort, anticipated benefits, and personal information protection. When drafting the consent form, the study’s contents and procedures were thoroughly explained to the parents of the patient in a quiet and stable independent space, allowing for sufficient time (at least 30 min). The collected personal information was strictly managed in compliance with relevant laws and regulations, with only authorized personnel having access to the data. Patient-identifying records were kept confidential, even when publishing the results of the clinical trials. During the sample analysis period, subject identification codes or clinical trial registration numbers were used, ensuring the diligent management of sensitive information to avoid exposure and anonymization (removal of personally identifiable information such as names, hospital registration numbers, and contact numbers) in accordance with bioethics and safety laws. Both a consent form for human biological material research and a donation consent form (Korea National Institute of Health and National Central Human Resources Bank) were necessary.

### 2.3. Development of Clinical and Epidemiological Information Collection Protocol (Case Record Form)

Demographic and clinical data from CA patients and their parents were collected. In addition, phenotype information according to the Human Phenotype Ontology (HPO) term and major test findings were recorded.

To gather information on environmental factors associated with the occurrence of CA, a questionnaire and a case record form were developed, (Figure 1), which assessed maternal and paternal exposure during and prior to pregnancy (Supplementary Tables S1–S4). Key items on this questionnaire included occupational history, exposure to hazardous substances in residential areas, medication intake, smoking, alcohol consumption, radiation exposure, increased body temperature, and cell phone use (Supplementary Tables S1–S4) [15]. For assessing exposure to fine particulate matter, modeling was utilized when an address was available. Consequently, additional items were included, such as the address of the residence for at least the past 2 years, actions taken during periods of high fine particulate matter concentration, and the use of air purifiers. Considering the prolonged periods of indoor residence during pregnancy, the questionnaire focused on indoor environments. After expert consultation, the final questionnaire items were approved and used to develop an electronic case report form.

### 2.4. Collection of Human Biospecimens and DNA Generation

Blood samples were collected from the study participants and their parents (ethylenediaminetetraacetic acid-treated tube). Parents also provided urine samples. These samples were processed to create research resources, including plasma, genomic DNA, and urine, which were stored in a −80 °C freezer for preservation. A total of 138 human biological resources, including plasma, genomic DNA, and urine samples, were obtained, as well as 138 sets of whole-genome sequencing data.

### 2.5. Generation of Genomic Information

Whole genome sequencing (WGS) was performed using blood samples from target infants and their parents. The library was prepared using the TruSeq Nano DNA Kit (Illumina, Inc., San Diego, CA, USA). Massively parallel sequencing was performed using a NovaSeq6000 with paired-end reads of 150 bp. FASTQ data were aligned to the human reference genome (hg19) using Burrows–Wheeler Alignment v0.7.17 [16]. Data preprocessing and variant calling were performed using the Haplotype Caller Genome Analysis Toolkit v4.2.0 [17]. Variants were annotated using ANNOVAR [18].

The samples have a mean depth of at least 30×, with more than 95% coverage of the human reference genome at more than 10×. At least 85% of the databases achieved a quality score of Q30 or higher. The donated data includes raw data (FASTQ), data processed using the reference genome (BAM), variant calling data (VCF), and a protocol electronic document that provides detailed instructions and commands for third-party analysis, ensuring data analysis reproducibility. The results will be reported in separate research papers.

### 2.6. Establishment of a Genetic Diagnosis Platform by the Multidisciplinary Expert Panel

In this study, we established a multidisciplinary expert panel comprising neonatologists, clinicians from diverse specialties, pathologists, geneticists, and members of the genetic laboratory. This panel interpreted the results obtained from WGS trios and took decisions regarding the need for additional genetic diagnostic tests, such as Sanger sequencing, RNA analysis, or functional studies, as necessary. Furthermore, we implemented a system for communicating genetically diagnosed cases to their parents formally through official reports. Moreover, we provided genetic counseling, whenever required, to facilitate comprehensive discussions about the results.

## 3. Discussion

Early diagnosis of CA in newborns is often challenging owing to various factors. The complexity of diseases accompanied by multi-organ deformities, as well as the ambiguity of symptoms, makes it difficult to differentiate them from other disease categories, including metabolic diseases. However, early diagnosis is essential to prevent ongoing organ damage and ensure optimal long-term growth and development in children. Treating CA in newborns requires multidisciplinary cooperation, involving close collaboration and specialized surgical treatment by experts in each field, as well as conservative treatment that considers nutritional support and newborn growth and development. In certain cases, patients may need to be referred to institutions offering more professional treatment. Genetic testing, including WGS and result interpretation, which are essential for genetic diagnosis in CA patients, may not be available in all hospitals. Establishing a social cooperation system that connects institutions capable of precise genetic testing and appropriate treatment is vital for selecting suitable patient groups, enrolling families in studies, and efficiently and safely collecting samples. This underscores the need for standardized protocols to collect clinical and genomic data for neonatal CA patients. By utilizing appropriate protocols and platforms for information collection, critically ill newborns can benefit from advanced infrastructure from birth, enabling early diagnosis and effective treatment [19].

Recent studies have utilized NGS to investigate birth defects and genetic mutations in newborns. The primary challenge lies in identifying novel or potential neonatal mutations that can account for the observed phenotypes. Parent-child trio WGS analysis can be a powerful approach to identifying non-inherited mutations; however, bias can complicate the identification [20].

Continuously updating the patient’s phenotype and genomic information is crucial and should be an area of ongoing supplementation and development in the future utilizing the foundational protocols presented in this study. In order to record and collect major phenotypes, symptoms, and significant examination findings, HPO terms should be used as much as possible for diagnosis and phenotype information. The CA registry observed newly developed or changing symptoms over time, along with continuous monitoring of growth and development indicators. Including additional items such as growth and development tests and conducting long-term follow-up on body measurements (weight, height, and head circumference) based on corrected age, K-DST, Bailey’s developmental test (in case of neurological abnormalities), visual acuity, hearing, readmissions, surgical procedures, and mortality would hold significant value [21]. Most importantly, novel genetic information that can elucidate phenotypes and diseases is continuously updated over time.

Through these two projects, a standardized CA patient registry was established, and protocols necessary for collecting clinical research resources for human biospecimen banking were developed. An important aspect of developing a research consent form is to include essential information regarding consent for the secondary use of resources within the preservation period after human biospecimen banking, and the inclusion of personally identifiable information. The target patients in this project were newborns with multiple major CA, all of whom had negative findings in all items and provided consent from both parents and family members to participate in the research. Even if the results of previous single-gene tests or genetic panel tests are positive, it would be meaningful to include infants with multiple CA that cannot be explained medically based on the phenotype for participation in the study.

Using the protocol developed in this study, future large-scale multicenter studies can be conducted to determine the causal relationship between specific environmental risk factors and CAs. It is necessary to accurately collect information such as the occupation of the mother and father, work environment, residential and living environment, exposure to teratogenic substances, medication intake, smoking history, alcohol consumption, caffeine, radiation exposure, heat increase, and dietary habits in the environmental questionnaire. Exposure to harmful environmental factors (i.e., medicines, smoking, alcohol, and chemicals) for more than six months, not only during delivery but also prior to exposure, was investigated.

The study has some limitations to be considered. The clinical and genomic information was collected in a medical center, which is not representative of the entire population. The protocol may require some modifications and supplements while conducting large-scale research. Evaluating the accuracy and robustness of these protocols will be required through further analysis in the future.

Based on the protocols developed through these two research and development projects and the secured human resources, a foundation for establishing the basic process of early genetic diagnosis of CA after birth and a practical clinical research platform for elucidating the genetic and environmental risk factors as causes of CA have been established. Specifically, WGS analysis of CA newborn–parent trio, obtaining essential consent forms based on the registry, developing a clinical information collection protocol using HPO terms as an objective tool for phenotypic quantification, introducing the basic concept of continuously updating clinical symptoms that may be newly added or changed over time, and developing a protocol that enables long-term tracking by adding new items that reflect the important characteristics of newborns, such as growth and development tests, have significant and innovative implications.

On the other hand, the present study does not intend to assess the diagnostic effectiveness of trio WGS or directly compare it with conventional methods. Instead, it focuses on establishing a standardized protocol for genetic diagnosis systems for infants with multiple CAs facing the practical challenges associated with implementing WGS in clinical settings, including technical accessibility, complex infrastructure, and the need for expert personnel [22]. However, it is crucial to emphasize that estimating diagnostic efficacy is highly important for assessing the utility of this genetic diagnostic system in real clinical settings and for its potential expansion into larger multicenter studies.

It is known that the traditional diagnostic methods typically achieve genetic diagnoses in approximately 20% of severely ill pediatric patients [23]. Whole exome sequencing (WES) generally exhibits a diagnostic rate of 20–40%, WGS has a rate of 40–60% [24,25,26]. A retrospective study found a diagnostic rate of 40% for NGS in newborn infants suspected of underlying genetic or metabolic conditions in neonatal intensive care units [27]. Another retrospective study reported a diagnostic yield of 36.7% for clinical WES in critically ill infants with suspected monogenic disorders in neonatal and pediatric intensive care units [28]. Additionally, the diagnostic rate of WES in individuals mostly adults with previously undiagnosed multiple malformation syndrome was 43.2% [29]. In another prospective study targeting acutely ill infants with suspected genetic diseases, including congenital anomalies, early WGS (within 2 months of age) achieved a diagnostic rate of 31.0%. Instead, the aggregate diagnostic rate for usual-care tests was 15.0%, however, it increased to 38.8% with the return of WGS results after 2 months of age. Notably, the largest number of diagnoses were made in infants with multiple congenital anomalies at 33% in that study [30].

Taking these findings into account, given that our present study is limited to infants with multiple CAs and WGS is conducted as a second-tier test following conventional studies, it is reasonable to expect a genetic diagnostic rate of approximately 20–40% for trio WGS. The results will be published later in the future.

## 4. Conclusions

The valuable information from the collected family trio’s clinical, epidemiological, and genomic information and protocols can directly contribute to early genetic diagnosis, identification of genetic and environmental risk factors, and personalized treatment for each patient’s CA. Building upon the registration method and protocol developed in this study, we propose a comprehensive plan to fully utilize and expand the study’s results on a national scale.

## Data Availability

Not applicable.

## Supplementary Materials

The following supporting information can be downloaded at: https://www.mdpi.com/article/10.3390/children10101673/s1, Table S1: Environmental factors questionnaire (maternity and spouse occupational history), Table S2: Environmental factors questionnaire (residence and living environment), Table S3: Caffeine, radiation, increased heat, and eating habits, and Table S4: Questionnaire on the exposure to deformity-causing substances.

## Abbreviations

CA	Congenital anomalies
NIH	National Institutes of Health
NGS	Next-generation sequencing
SMC	Samsung Medical Center
HPO	Human Phenotype Ontology
DNA	Deoxyribonucleic acid
WGS	Whole genome sequencing
RNA	Ribonucleic acid
WES	Whole exome sequencing
