# Supplementary material for: Optimal Protocols and Management of Clinical and Genomic Data Collection to Assist in the Early Diagnosis and Treatment of Multiple Congenital Anomalies
Source: Children (Basel). 2023 Oct 10;10(10):1673. doi: 10.3390/children10101673 (PMC10605914; doi:10.3390/children10101673)
Supplement: Supplementary file 1 [file children-10-01673-s001.zip › Supplementary Table S1.pdf]

Table S1. Environmental factors questionnaire (maternity and spouse occupational history).

Environmental Factors Questionnaire (Maternity Occupational History)

I have an occupation where I've worked for more than 6 months	◯ Yes ◯ No
Select, If Yes	:::Select if yes:::
If Other, Describe your occupation	

I handled chemical substances during my workday.	◯ Yes ◯ No<br>Excluding handling general office material.

Chemical Substances

Chemical Substances	Usage Status	Frequency of Use	Period of usage	Usage Method
Paint, Lacquer, Varnish	◯ Yes ◯ No<br>Answer this row only if you used them	day(s)/week<br>How many days a week?<br>hours/day<br>How many hours a day?	From ________to ________	◯ Brush ◯ Roller ◯ Spray ◯ Other<br>How did you use it?
Glue	◯ Yes ◯ No<br>Answer this row only if you used it	day(s)/week<br>How many days a week?<br>hours/day<br>How many hours a day?	From ________to ________	◯ Metal ◯ Wood ◯ Plastic ◯ Other<br>What did you glue?
Cleaning agents and degreasing agents	◯ Yes ◯ No<br>Answer this row only if you used them	day(s)/week<br>How many days a week??<br>hours/day<br>How many hours a day?	From ________to ________	Metal ◯ Plastic ◯ Other<br>What did you clean?<br>◯ Cotton ◯ Spray ◯ Tank ◯ Others<br>How did you clean/degrease?
Paint removers	◯ Yes ◯ No<br>Answer this row only if you used them	day(s)/week<br>How many days a week?	From ________to ________	◯ Metal ◯ Wood ◯ Other<br>What did you remove?
Other organic solvents	◯ Yes ◯ No<br>Answer this row only if you used them	day(s)/week<br>How many days a week?<br>hours/day<br>How many hours a day?	From ________to ________	
Do you wash your hands with chemicals other than commercial soaps or hand sanitizers?	◯ Yes ◯ No<br>Answer this row only if you used them			

Other	
