# Supplementary material for: Optimal Protocols and Management of Clinical and Genomic Data Collection to Assist in the Early Diagnosis and Treatment of Multiple Congenital Anomalies
Source: Children (Basel). 2023 Oct 10;10(10):1673. doi: 10.3390/children10101673 (PMC10605914; doi:10.3390/children10101673)
Supplement: Supplementary file 1 [file children-10-01673-s001.zip › Supplementary Table S2.pdf]

Table S2. Environmental factors questionnaire (residence and living environment).

Environmental Factors Questionnaire (Residence and Living Environment)

- Within 50m: Within 1 minute walk
- Within 100m: Within 8-minute walk
- Within 500m: More than 8-minute walk

**Was there a 4-lane road near your house during pregnancy?**	○ Yes ○ No				
**If yes, select the distance to the road**	I don't know.	within 50 meters	within 100 meters	within 500 meters	within 1km

**Are there any of these following facilities withing 2km near your house during pregnancy?**	○ Garbage Incinerator ○ Landfill ○ Sewage Treatment Plant<br>○ Factories (Handling Chemicals, Tires, Coal, Pesticides, Fertilizers, etc.)<br>○ Bus Terminals/ Taxi Dispatchers ○ Chemical Treatment Plant<br>○ Crematorium<br>○ Other ○ Not Applicable ○ I don't know.	
**If Other, Describe in detail**		
**Are you ventilating your house?**	○ Yes ○ No	
**If yes, which ventilation method do you use?**	☐ Opening the windows	time(s) /day
	☐ Local ventilation facilities (air purifier, ventilation fan)	
	☐ Central ventilation system	
	☐ Other	
	☐ I don't know.	

	**Usage Status**	**If yes, how long is the usage period?**	
**Are you using an air purifier during pregnancy?**	○ Yes ○ No	day(s)/week<br>How many days per week?	hours/ day<br>How many hours per day?
**Are you using a humidifier during pregnancy?**	○ Yes ○ No	day(s)/week<br>How many days per week?	hours/ day<br>How many hours per day?
**If yes, are you using a cleaner?**	○ Yes ○ No		
**Are you using an air conditioner during your pregnancy?**	○ Yes ○ No	day(s)/week<br>How many days per week?	hours/ day<br>How many hours per day?

**Have you moved to a newly built or renovated house in the last year?**	○ Yes ○ No
