# Supplementary material for: Optimal Protocols and Management of Clinical and Genomic Data Collection to Assist in the Early Diagnosis and Treatment of Multiple Congenital Anomalies
Source: Children (Basel). 2023 Oct 10;10(10):1673. doi: 10.3390/children10101673 (PMC10605914; doi:10.3390/children10101673)
Supplement: Supplementary file 1 [file children-10-01673-s001.zip › Supplementary Table S3.pdf]

Table S3. Caffeine, radiation, increased heat, and eating habits.

Caffeine Intake

**Coffee**	○ None	Once or twice a day	3 to 5 times a day	6 to 7 times a day	8 times or more a day	a week/times
**Green tea**	○ None	Once or twice a day	3 to 5 times a day	6 to 7 times a day	8 times or more a day	a week/times
**Coca-Cola, Pepsi**	○ None	Once or twice a day	3 to 5 times a day	6 to 7 times a day	8 times or more a day	a week/times

Radiation Exposure Status

**Radiation exposure status**	○ Yes ○ No
**Timing of radiation exposure**	○ Before 10 weeks of pregnancy (counting from the first day of your last menstruation)<br>○ Between 10 and 15 weeks of pregnancy ○ Between 16 and 25 weeks of pregnancy<br>○ After 26 weeks of pregnancy ○ Other
**If other, select the following**	

**Chest X-ray**	○ Yes ○ No
**Abdominal X-ray**	○ Yes ○ No
**Breast X-ray**	○ Yes ○ No
**Isotopes of the thyroid gland**	○ Yes ○ No
**Jugular sinusography**	○ Yes ○ No
**Photographing large and small intestines with barium**	○ Yes ○ No

**Head CT**	○ Yes ○ No
**Chest CT**	○ Yes ○ No
**Abdominal CT**	○ Yes ○ No
**Lumbar CT**	○ Yes ○ No
**Pelvic CT**	○ Yes ○ No

Experience Regarding Increasing Temperature

**Thermal experience**	○ Yes ○ No ○ I don't know

	**Thermal Increase Status**	**Timing of the Experience**	**Total Number of Experiences (Times/Week)**
**Sauna**	○ Yes ○ None	• Before 5 weeks of pregnancy (counting from the first day of your last menstruation)<br>• Between 5 to 10 weeks of pregnancy<br>• Between 11 to 20 weeks of pregnancy<br>• After 20 weeks of pregnancy	
**Hot bath**	○ Yes ○ None	• Before 5 weeks of pregnancy (counting from the first day of your last menstruation)<br>• Between 5 to 10 weeks of pregnancy<br>• Between 11 to 20 weeks of pregnancy<br>• After 20 weeks of pregnancy	

**High fever**	◯ Yes ◯ None	• Before 5 weeks of pregnancy (counting from the first day of your last menstruation)<br>• Between 5 to 10 weeks of pregnancy<br>• Between 11 to 20 weeks of pregnancy<br>• After 20 weeks of pregnancy	

**Comments**	

Eating Habits Questionnaire (Basic Information on Eating Habits)

**Number of meals/day**	◯ Once Twice 3 times More than 4 times
**Are you a vegetarian?**	Yes, I am No, I am not
