# Supplementary material for: Optimal Protocols and Management of Clinical and Genomic Data Collection to Assist in the Early Diagnosis and Treatment of Multiple Congenital Anomalies
Source: Children (Basel). 2023 Oct 10;10(10):1673. doi: 10.3390/children10101673 (PMC10605914; doi:10.3390/children10101673)
Supplement: Supplementary file 1 [file children-10-01673-s001.zip › Supplementary Table S4.pdf]

Table S4. Questionnaire on the exposure to deformity-causing substances.

Smoking History

Exposure to deformity-causing substances questionnaire (smoking history)

**Smoking status**	○ Non-smoker (Never smoked)<br>○ Former smoker (Smoked before but quit before or during pregnancy)<br>○ Current smoker				
**If you're a 'Former smoker', select when you quit smoking**	6 weeks before pregnancy	6 to 10 weeks of pregnancy.	11 to 20 weeks of pregnancy	After the 20th week of pregnancy	Other
**Number of cigarettes per day for 'Former smoker' and 'Current smoker'**	Less than 5 cigarettes	6 to 7 cigarettes	One pack	2 packs	

**Second-hand smoke status**	○ Yes ○ No<br>It refers to second-hand smoke indoors or in an enclosed space (Excluding second-hand smoke executed outside walking, streets, buses/taxi stations).				
**Second-hand smoke frequency**	Every day	4-6 times a week	2-3 times a week	Once a week	I don't know

Drinking history

Exposure to deformity-causing substances questionnaire (drinking history)

**Drinking status**	○ Non-drinker<br>○ Former drinker (Drank before but quit before or during pregnancy)<br>○ Current drinker
**If you are a 'Former drinker', when you stopped drinking**	○ 6 weeks before pregnancy ○ 6 to 10 weeks of pregnancy ○ 11 to 20 weeks of pregnancy<br>○ After the 20th week of pregnancy ○ Before pregnancy
**Drinking frequency**	○ 2-3 times a month ○ once or twice a week ○ 3 to 4 times a week<br>○ 5 to 6 times a week ○ every day
**Average alcohol intake in the case of 'former drinker' and 'current drinker'**	○ 1-2 shots of soju, 2 shots of strong liquor, less than 1 ½ 355cc can of beer<br>○ 3-4 shots of soju, 4 shots of hard liquor, 3 355cc cans of beer<br>○ 5-6 shots of soju, 6 shots of hard liquor, less than 4 ½ 355cc cans of beer<br>○ 7-9 shots (less than 1 ½ bottle) of soju, 9 shots of hard liquor, 6 355cc cans of beer<br>○ More than 10 shots (more than 1 ½ bottles) of soju, 10 shots of hard liquor, 7 355cc cans of beer
